# Intelligence

**Published:** 1830-07

**Authors:** 


### INTELLIGENCE.

### ROYAL WESTMINSTER OPHTHALMIC HOSPITAL.

***Report of the Committee of Management for the Year ending 31st May, 1830.***

**DURING the last year 1624 persons, of all ages, have been admitted to the benefits of the charity, making a total of 16,895 since its institution. Of these 550 have been restored to sight by the different operations for cataract, some who have been born blind, and some at a very advanced period of life, and after many years of blindness. Several of these, old soldiers and sailors discharged from the public service, after having wandered through the streets in a state of destitution, have been rendered comfortable and comparatively happy through this charity, which, from the nature of its foundation, is always open for their reception.**

**Your Committee have reason to believe that, under the improved methods of treatment introduced at this hospital within the last three years, those terrible diseases which have often afflicted our fleets and armies, and distressed the poor in workhouses and infant-asylums, will be suppressed as soon as they may appear.**

**The Committee, in the report of last year, gave the names and addresses of sixteen children, blind from a disease acquired in their birth, which is always curable, if immediate application is made for assistance, or if the nature of it be generally understood. The present Committee regret to add a list of twenty more irrecoverably blind of one or both eyes, for want of a little assistance, which, if extended through the medium of this hospital during the first weeks of their existence, would have secured them against the greatest of misfortunes.**

**[Here follow the names and residences of the patients referred to.]**

**The Committee earnestly wish to impress on the public, that another and most important benefit to mankind arises from this hospital. Its offering the means of instruction to medical students, by which they may be enabled again to extend the advantages derived from this charity in every direction; and the course of Lectures on the Principles and Practice of Surgery, and on the Diseases of the Eye, given by your surgeon during the winter season, is open gratuitously to all medical officers of the navy, the army, the ordnance, and the East India Company, through whom the greatest benefits have been conferred in various quarters of the globe to an extent scarcely to be believed.**

**In order to be enabled to receive the poor and often destitute persons who apply for advice, a piece of ground has been rented in Chandos street, near the Strand, on a lease for 99 years, from the Commissioners of Woods and Forests, for the erection of an hospital capable of containing from twenty to thirty in-patients, who will be received from all parts of the country, on the recommendation to the Committee of the mayor, magistrates, resident clergymen, or by a letter from any of the subscribers to the hospital. The sum of £5000 is considered sufficient to effect all the objects in contemplation. Of this £2500 have been subscribed, and the Committee rely on the humanity and charity of the public for the remainder, and for the future support of the hospital.**

**By order of the Committee,**

**W. T. HUGHES, *Secretary.***

*Prospectus of the "Metropolitan Society of General Practitioners in Medicine and Surgery."*

WHILE almost all public bodies, whether professional or commercial, form associations, corporations, or companies, for the purposes of legislating for their mutual protection and for the advancement of their prosperity, it is found that no association of the numerous class of medical men comprehended under the term General Practitioners, has in any manner been yet formed for the protection of their particular interests.

Various branches of the medical profession have colleges, charters, and corporations, from which the general practitioner is either altogether excluded, or attached as an appendage only; he is not admitted to a participation in their *Councils*, or to share in their honours; as a general practitioner, he belongs exclusively to no one branch, and is, therefore, virtually excluded from all.

A society has, therefore, been formed, entitled "The Metropolitan Society of General Practitioners in Medicine and Surgery," which is intended as an union of the practitioners of this class throughout England and Wales, for the protection of their mutual and individual interests; having the following objects:

1st. Such alteration of existing laws and customs as shall promote the prosperity and respectability of the general body of practitioners.

2d. The adoption of such measures as may be conducive to the advancement of medical science, and of professional information.

3d. The periodical assembling of the members for literary and scientific discussion; for the cultivation of social intercourse, and for the consideration of general measures relative to the Society.

4th. The creation of a fund to be appropriated to the protection of the members, and for the general exigencies of the Society.

5th. The establishment of a benevolent fund, by contributions from members of the profession at large, and other charitable persons, for the relief of distressed medical men and their families.

The limits of a prospectus will not allow of a full detail of the objects contemplated; but it may be observed, in addition to the foregoing general statement, that it is intended, as soon as practicable, to effect some regulation respecting the mode of professional compensation; and, if necessary, to procure a legislative enactment to authorise the general practitioner to make a fair and open charge for his services. It it also intended to protect, individually, those members who may become involved in questions which may be considered by the committee to affect the interests of the Society as a body.

Notwithstanding that there are numerous charitable funds for relieving distressed members of particular branches of the medical profession, it is found that there are many members of that profession who are not objects of relief from any of those funds; and it is, therefore, to supply this desideratum, that the plan of a General Benevolent Fund has been adopted, the application of which, it is intended, should not be confined to this Society exclusively, but should be extended, at the discretion of the committee, to every member of the profession.

The affairs of the Society are under the management of a president, vice president, and a committee.

A house, or chambers, will be engaged, as early as possible, for the use of the Society.

The Society will meet at such stated periods, and in such manner, as will be hereafter determined.

The foregoing is a brief statement of the views of the founders of this Society, and of the advantages intended from its institution, the plan of which may be enlarged, or curtailed, according to the support it may receive.

The committee of management entertain a confident hope that the Society will be of great utility to the general body of practitioners, whose attention to this subject is earnestly recommended.

WILLIAM GAITSKELL, *President.*

It is requested that all applications and communications be made and addressed (post paid) to MR. W. SENHOUSE GAITSKELL, solicitor, 21, Stamford street, Blackfriars.

### METEOROLOGICAL JOURNAL,

By Messrs. Harris and Co. Mathematical Instrument Makers, 50, High Holborn.

| May | Moon. | Rain gauge. | Thermom. 9 a.m. | max. | min. | Barometer. 9 A.M. | 10 P.M. | De Luc's Hygrom. 9 A.M. | 10 P.M. | Winds. 9 A.M. | 10 P.M. | Atmospheric Variations. 9 a.m. | 2 p.m. | 10 p.m. |
|---|---|---|---|---|---|---|---|---|---|---|---|---|---|---|
| 20 | | | 66 | 70 | 55 | 29.81 | 29.72 | 44 | 45 | SSW | E | Fine | Fine | Fine |
| 21 | | | 59 | 63 | 50 | .69 | .62 | 45 | 46 | E | E | —— | —— | —— |
| 22 | ● | | 60 | 63 | 56 | .70 | .80 | 57 | 55 | W | NW | Cloudy | —— | —— |
| 23 | | | 62 | 68 | 52 | .67 | .58 | 59 | 65 | NE | NE | Fine | —— | —— |
| 24 | | | 60 | 63 | 53 | .52 | .58 | 66 | 61 | SSE | SSE | Cloudy | Cloudy | —— |
| 25 | | | 65 | 69 | 53 | .50 | .37 | 56 | 55 | WSW | SW | Fine | Fine | Rain |
| 26 | | | 57 | 62 | 51 | .29 | .34 | 55 | 55 | W | W | Cloudy | Rain | Cloudy |
| 27 | | .134 | 58 | 55 | 50 | .35 | .46 | 55 | 53 | W | WNW | Cloudy | Cloudy | Cloudy |
| 28 | | | 53 | 60 | 49 | .69 | .83 | 53 | 52 | WNW | WNW | —— | —— | —— |
| 29 | ☽ | | 59 | 64 | 52 | .93 | .84 | 52 | 49 | NW | WSW | Fine | Fine | Rain |
| 30 | | | 57 | 61 | 50 | .62 | .68 | 52 | 54 | | | Rain | Rain | Fine |
| 31 | | .45 | 59 | 61 | 55 | .72 | .85 | 55 | 54 | SW | SW | Cloudy | Cloudy | Cloudy |
| June | | | | | | | | | | | | | | |
| 1 | | | 60 | 65 | 51 | .90 | .94 | 54 | 54 | SW | WSW | Cloudy | Fine | Fine |
| 2 | | | 62 | 62 | 54 | 30.00 | .92 | 54 | 54 | WSW | WSW | Fine | —— | —— |
| 3 | | | 62 | 62 | 51 | 29.69 | .40 | 54 | 61 | NE | SSW | Cloudy | Rain | Cloudy |
| 4 | | .37 | 56 | 60 | 51 | .43 | .75 | 65 | 62 | SW | WSW | Cloudy | Cloudy | Cloudy |
| 5 | | | 54 | 61 | 59 | .87 | .85 | 56 | 54 | WSW | SW | Fine | Fine | Fine |
| 6 | ○ | | 64 | 71 | 55 | .81 | .78 | 55 | 55 | W | W | —— | —— | —— |
| 7 | | | 58 | 63 | 53 | .76 | .76 | 56 | 55 | SW | NW | Rain | Fine | Cloudy |
| 8 | | | 56 | 62 | 51 | .78 | .91 | 55 | 57 | NW | NNW | Cloudy | —— | Fine |
| 9 | | | 54 | 57 | 50 | .92 | .91 | 60 | 61 | N | NNE | Cloudy | Rain | Show'ry |
| 10 | | .27 | 52 | 58 | 51 | .86 | .83 | 63 | 62 | NW | NW | Cloudy | Rain | Cloudy |
| 11 | | | 58 | 59 | 55 | .83 | .79 | 61 | 64 | WNW | WNW | Cloudy | Fine | Cloudy |
| 12 | | .24 | 58 | 63 | 52 | .61 | .65 | 54 | 56 | WSW | NW | Sleet | Fine | Cloudy |
| 13 | ☾ | | 56 | 62 | 50 | .60 | .57 | 56 | 54 | W | W | Fine | Cloudy | Cloudy |
| 14 | | .12 | 53 | 61 | 48 | .53 | .45 | 53 | 54 | WNW | WNW | Cloudy | Show'ry | Rain |
| 15 | | | 51 | 56 | 49 | .55 | .55 | 55 | 57 | NNE | NNW | Show'ry | Rain | Cloudy |
| 16 | | .54 | 55 | 52 | 42 | .48 | .70 | 58 | 56 | W | NNE | Sleet | Fine | Rain |
| 17 | | 106 | 50 | 58 | 42 | .77 | .79 | 56 | 58 | N | N | Cloudy | Fine | Cloudy |
| 18 | | | 58 | 62 | 50 | .68 | .52 | 58 | 56 | NNW | WSW | Cloudy | Fine | Fine |
| 19 | | | 59 | 65 | 48 | .47 | .52 | 50 | 50 | WNW | W | Fine | Fine | Fine |

The quantity of Rain fallen in May, was 2 inches and 29-100ths.

